# Building health systems capacity in global health graduate programs: reflections from Australian educators

**DOI:** 10.1186/1472-698X-12-14

**Published:** 2012-08-24

**Authors:** Joel Negin, Alexandra Martiniuk, Chris Morgan, Philip Davies, Anthony Zwi

**Affiliations:** 1 Senior Lecturer in International Public Health, School of Public Health, University of Sydney, Edward Ford Building (A27), Sydney, NSW, 2006, Australia; 2 Senior Research Fellow, George Institute for Global Health, and Senior Lecturer, University of Sydney, Sydney, Australia; 3 Associate Professor, Dalla Lana School of Public Health, University of Toronto, Toronto, Canada; 4 Principal Fellow, Burnet Institute, Adjunct Senior Lecturer, Monash University School of Public Health and Preventive Medicine, Honorary Fellow, University of Melbourne School of Population Health, Melbourne, Australia; 5 Professor of Health Systems & Policy, School of Population Health, University of Queensland, Herston, Australia; 6 Professor of Global Health and Development, School of Social Sciences and International Studies, University of New South Wales, Sydney, NSW, Australia

## Abstract

There has been increasing focus on the role of health systems in low and middle-income countries. Despite this, very little evidence exists on how best to build health systems program and research capacity in educational programs. The current experiences in building capacity in health systems in five of the most prominent global health programs at Australian universities are outlined. The strengths and weaknesses of various approaches and techniques are provided along with examples of global practice in order to provide a foundation for future discussion and thus improvements in global health systems education.

## Correspondence

The role of health systems in improving population health in less developed countries is increasingly attracting attention [1,2]. Strengthening health systems has come to be seen as a prerequisite to achieving the Millennium Development Goals [3,4]. Most global health agencies, including the World Health Organization [5], USAID [6] and the World Bank [7], now put health systems at the centre of their health strategies. Disease-specific global health initiatives increasingly link their objectives and funding to health system strengthening [8]. This has led to the assertion that: “a consensus is growing about the need for greater global action on health systems, representing a new phase in global health policy” [9].

Health systems research and teaching engage with an array of fields examining issues of cost, quality, accessibility, delivery, organisation and financing and how they interact to improve health outcomes [10]. The WHO health systems building blocks [5] are the most commonly used framework for understanding health systems along with the ‘control knobs’ for health system reform [11] and the Systems Framework [12]. Most frameworks acknowledge the importance of core systems areas including the health workforce, financing, information systems, service delivery and governance which together can lead to the achievement of better health status.

Despite the increasing focus on health systems, there remains considerable debate about what constitutes health systems [13] and health systems strengthening [14,15]. Some advocate viewing health systems as ‘complex adaptive systems’ [16] to incorporate the dynamic, multiple influences on health services. Most commentators now call for contributions from disciplines including economics, management, anthropology, sociology and policy analysis [4]. The lack of a common understanding and set of guiding principles remains a challenge leading to warnings that health systems could become a meaningless “buzzword” [8].

## Public health teaching and health systems

A recent review of health sciences education confirms the paucity of insights on public health education. Frenk et al. found that of 11,054 publications on the education of health professionals, 73% were about medical education and only 2% covered public health – much less health systems [17]. Evans asserts that “for an evidence-based discipline, it is surprising that there has been little research or reflection on [the role of schools of public health in tackling global public health challenges] within the public health literature” [18] and Sadana and colleagues confirm that “there is almost no evaluation of the degree to which education practices match real-world dimensions” [19].

National policy makers have a very high demand for health systems experts in financing, policy, and management [20]. Given the importance and complexity of health systems, teaching about developing country health systems should be widely and rigorously conducted in global health and masters of public health (MPH) programs especially since these programs are often the pathway of training for health professionals moving into health leadership roles. But, as Frenk et al. note, the education of health professionals “has not kept pace” with emerging challenges due to “outdated and static curricula” and is not preparing graduates for the broad, multi-context environment in the real world [17]. Capacity development in health systems has been neglected [21] leading to a shortage of people skilled in the area [22]. Most schools of public health adhere to traditional public health education approaches emphasising epidemiology, biostatistics, communicable diseases and health promotion [23]. Despite this, a MPH remains one of the most common qualifications of senior health managers.

At the same time, there has been a marked increase in global health teaching. The number of undergraduate, graduate and doctoral students enrolled in global health programs in the US doubled between 2006 and 2009 [24]. The North America-based Consortium of Universities for Global Health now has 53 members.

With this in mind, Frenk et al. call for a redesign of professional health education with a focus on systems-based instruction [17]. From the literature, there is no clear consensus on the competencies needed for global health and for health systems specifically [21]. Merson interviewed policy makers in developing countries to identify the health system competencies in greatest need; these included resource allocation, analysing health systems policy issues, strategic planning, human resource management and conducting cost-effectiveness analyses [20]. More recently, the Association of Schools of Public Health in the US have started developing a set of competencies for global health which include comparative health systems analysis, economic analysis and context-specific policy making processes [25].

There have also been efforts to identify competencies for Australian health professionals working in global health [26]. This predated the current emphasis on systems, but nevertheless highlighted basic public health skills such as disease control and prevention alongside communication and cross-cultural skills, management and planning competencies. The ability to analyse or plan health systems did not receive deeper attention although the paper noted that the “increased emphasis on health sector reforms placed new demands” on public health practitioners. The authors argued that “a better understanding of health sectors” was needed, but little was proposed to address this gap [26].

We have also engaged in discussions with potential employers in Australia and the region about skill shortfalls and training needs for their staff. The draft Pacific health strategy currently being developed by the Australian Agency for International Development notes the need to increase staff capacity to engage in the complexities of health systems and practice and the World Bank Pacific Office actively provides short courses on health financing to Pacific Island health officials to strengthen their health financing skillsets. These efforts highlight the need for more capacity building in health systems in the region.

## The Australian experience in teaching health systems

A number of Australian public health programs have taken steps to address the shortfall in health systems teaching from a global perspective. We describe and analyse the Australian experience of teaching about health systems focused on developing countries in a number of the most prominent public health academic programs in Australia. When reviewing the programs, we aimed to address the following core issues: a) what types of courses included health systems components focused on developing countries and what content did they include; b) what pedagogical methods were employed; c) which approach to understanding health systems was adopted.

In 2009, the University of Sydney’s Masters of International Public Health program offered, for the first time, a course called “Health Systems in Developing Countries.” This course is structured around the WHO building blocks with case studies as well as a focus on health systems research. The unit endeavours to move beyond diagnoses of challenges to identify evidence-based solutions and to prepare students to analyse, evaluate and implement health system interventions and reforms. The course provides lectures addressing key LMIC health systems debates such as the role of community health workers in service delivery, migration of health professionals, the appropriateness of user fees in resource-poor settings, health insurance models and sector-wide approaches to health governance. Students are asked to develop their own health systems research questions for a developing country context and to propose a methodology for answering that question. Guest lectures are provided by World Bank and AusAID representatives and by practitioners involved in improving health systems.

The interest in the course was very high – especially from international students. About half of all students enrolled in the University’s Masters of International Public Health program opted to take the course as an elective, more than 65% of whom are from low and middle income countries. Students came from a wide range of backgrounds including: medicine, nursing, political science, law and management. In student evaluations, 97% of students stated the unit was relevant and 100% rated the content as ‘excellent’ or ‘good’.

In addition, the University of Sydney offers a Masters of Health Policy that focuses on domestic health reform issues but includes international examples and a course on “Global Health Policy.” This program emphasises political science, policy processes and economic evaluation.

The University of Queensland (UQ) offers, through its School of Population Health, a postgraduate course on ‘Health Systems’ which is compulsory for all students studying for a Masters of Public Health. The content encompasses most aspects of health systems but does not have a primary global health focus. Lectures on health systems topics including financing and human resources, address issues that are directly relevant to less developed countries whereas other lectures focus on issues which are of more direct domestic concern (such as Aboriginal and Torres Strait Islander health). The course typically attracts between 80 and 120 students per semester, with approximately 50% international students. The UQ MIPH, for students interested in global health, does not have a specific course on health systems but the topic is mentioned in several courses.

Beyond the School of Population Health, the UQ Faculty of Business, Economics and Law offers a Masters of Health Economics program with an option to specialise in ‘Health and Development’. While the program draws heavily on Population Health courses, it also includes courses on ‘Globalisation and Economic Development’ and ‘Health & Economic Development’. To the extent that such a course might be considered as relevant to health systems it raises questions about the natural academic ‘home’ for such teaching.

The University of Melbourne’s masters level global health systems teaching is integrated within other global health subjects. A core component within the global health stream of the MPH is the subject ‘Primary Health Care in Developing Countries’ offered (unusually) as a subject co-accredited with Monash University and described below. An alternative is a residential study option “Primary Health Care, Jamkhed, India” which consists of a practical, experiential course offered for three weeks in central India. Other subjects relevant to global health integrate health systems concepts where relevant to the topic theme, notably “International Child Health” and “Public Health Leadership Management”. Other health systems elective subjects within the MPH are titled “Health Systems” and “Health Policy”, however these focus on the Australian health system rather than addressing global health system issues. This university, through the Nossal Institute of Global Health, has introduced a new subject, available to MPH students and other students interested in global health, in 2012. This is titled “Systems for Global Health” and aims to develop skills in using analytic tools, applying health system frameworks, and use of evidence in policy change for health system reform. The subject focuses on LMICs, their health and development context, health systems, health financing, use of financing to improve equity, and modes of influencing health policy.

Monash University has a diverse MPH program and a parallel Masters of International Health. The subject ‘Primary Health Care in Developing Countries’ offered through the Burnet Institute, acts as a primer on global health issues and incorporates health system frameworks, global health actors, health system strengthening approaches, and health financing options. It is compulsory for international health students. At Monash University, health systems are taught in a cross-cutting manner. MPH subjects integrate global health systems concepts appropriate to LMICs and relate these to the technical focus of the subject (for example, “Control of Communicable Disease in Developing Countries” or “Women and Children’s Health in Developing Countries”). Since 2011, the subject “Health Policy and Prevention in a Global World” has included global health system perspectives using the “control knobs” framework promoted by the World Bank and Harvard University. All these subjects attract a sizable cohort of international students and are among the most popular course offerings.

The University of New South Wales School of Public Health and Community Medicine offers a Masters of Public Health, Masters of International Public Health and Masters of Health Management. Health systems are integrated within many of the course units but are brought to the fore, for example, in the course on International Health. This course integrates health systems thinking and approaches, and highlights not only the policy dimensions, but the importance of equitable, responsive and efficient health systems. The course explores the use of evidence and the sensitivity of context, and considers also the different interests which operate within health systems and service delivery environments. Short courses on human resources, health and social aspects of disasters, and on maternal, neonatal and child health also highlight systems issues.

## Global practice in teaching health systems

The Australian experience is in keeping with international efforts to improve understanding and teaching of health systems. The London School of Hygiene and Tropical Medicine and Johns Hopkins Bloomberg School of Public Health each offer multiple courses in health systems focused on developing countries. The Harvard School of Public Health’s offerings focus on health sector reform and Columbia University’s Mailman School of Public Health recently added “Health and Health Systems in Low Income Countries” to their course offerings . Despite these examples, many public health programs do not have an explicit focus on global health systems.

A few schools of public health in middle-income countries have relatively substantial teaching on health systems. Programs in Brazil, Mexico, Bangladesh and South Africa in particular focus on health systems in their teaching programs. The Escola Nacional de Saúde Pública Sergio Arouca in Rio de Janeiro, Brazil for example, offer courses in health systems topics addressing building blocks such as management, service delivery, health information, and policy. The BRAC University School of Public Health in Bangladesh includes health system management and health care financing as core courses. South African schools of public health have also shown leadership in health systems education. The University of Pretoria, for example, demonstrates the importance it places on health systems by its name: the School of Health Systems and Public Health. Many others highlight strong areas of engagement with systems such as the University of the Western Cape with its strong human resources in systems orientation, as well as the Universities of Cape Town and Witwatersrand emphases on equity, resource allocation, policy analysis and evidence-informed decision-making.

## Lessons learned

Numerous Australian Universities have updated their educational offerings to include health systems in LMIC and to better prepare their students for a global health career. These experiences highlight the challenges for programs interested in improving their health systems capacity development.

Different models have been proposed at different Universities. The University of Sydney offers a unit directly focused on health systems and the WHO health systems building blocks, in order to devote greater time to the analytic tools and issues involved. The University of Sydney method allows for deep engagement in health systems issues but does run the risk of “verticalising” health systems so that human resources for health challenges are seen as an issue separate from wider health challenges. Monash University has integrated components of health systems thinking into its course offerings focused on specific technical themes, believing that all students of health issues in LMICs need some grasp of health systems in those settings. Integration means that the health system elements most emphasised are those relating to service delivery, technologies or human resources, rather than higher-order issues such as financing or governance. University of Melbourne has taken this integrated approach until 2012, after which it will also offer a unit focused on global health systems, albeit one that emphasises the health financing aspects of these. The UNSW integrates systems thinking and systems approaches into the course on International Health, using case based teaching methods to introduce system-relevant issues. For example, they unpack the various systems causes and effects of a child’s death in Timor-Leste as a case study in understanding health systems challenges in LMIC.

Whether health systems are better suited to be taught as a cross-cutting theme or as focused course(s) of its own, or both, is an important discussion point for educators. The cross-cutting approach might expose a wider range of students to health systems thinking while a specific course can provide additional depth and analytic tools. Even taking a cross-cutting approach, it has proven feasible to at least alert students to the range of frameworks available through with to analyse system issues. As the field of health systems matures, a corpus of frameworks and tools that cannot easily be taught in the limited time available to cross-cutting approaches is apparent. Indeed, it may well be that the use of such tools is better learned through workplace practice or other apprenticeship models, rather than in post-graduate educational programs.

The experience and skills needed to expertly teach health systems is another challenge for Australian Universities. Whereas capacity to teach other public health skills such as biostatistics and disease control currently exist in most public health schools, deep experience within developing country health systems is not as common. Given that the intense focus on health systems is relatively new, there is a limited cadre of experts in Australian educational institutions able to teach about health systems in developing countries. This highlights the likely value of interfacing with governments, civil society and international agencies, as this is where much of the experience resides.

Another dilemma is whether or not health systems in developing countries should be taught separately from domestic systems. While health systems frameworks endeavour to be universal, a number of the health systems bottlenecks and challenges in low and middle-income developing countries might be different from those of developed countries. Our review and experience shows that the conceptual frameworks and terminologies used by prominent global actors to describe health systems in LMICs vary significantly from those used to describe Australia’s health system. Health system issues such as financing, health workforce, and the role of external actors are likely to be distinct between developing and developed countries even if some lessons have value for both settings [27]. This is especially obvious in countries where overseas development assistance is a prominent source of health funding, such as the small island states neighbouring Australia.

Given the international nature of public health education, global health courses must prepare two types of student for service: those from developed countries seeking to work in LMICs, and those from LMICs returning to work in their countries. The former group need, in addition to a good understanding of health systems’ function and reform, to learn the practical skills and cultural competence to function effectively as ‘outsiders’ in LMICs. Both groups need to learn how to optimise the interaction between global health agencies (for example within the United Nations system or global health initiatives such as the Global Fund). It is difficult to convey such skills in standard academic course structures, although most courses described here attempt this through group work, problem-based learning, case scenarios and other practical exercises. Workplace attachments offer one avenue for such learning and in many universities may be incorporated into research theses.

This paper has focused on teaching of health systems in MPH and similar programs within Schools of Public Health. Given the emphasis on policy, management, cost analysis, information technology, and workforce within health systems, it is important to note that non-health specific programs teaching business administration or public policy provide complementary skills that are important to global health delivery. Perhaps health systems are so complex and diverse that the topic cannot – and perhaps should not – be fully taught within MPH curricula. The need for insights from international relations, finance, public administration and complex reform suggests that health systems could be promoted as an area of study and that it is worth considering building health systems capacity coursework within masters of business administration programs or Schools of Government [28]. Certainly, as a start, schools of public health should draw on expertise located in a much wider array of disciplines. Whether a successful leader in managing a health system requires a medical or MPH qualification at all remains a pertinent question.

## Summary

Bennett and colleagues cite “an urgent need” to build the health policy and systems field [22]. The need to integrate teaching on health systems into training programs is clear, yet the evidence on how this is being done, or should be done, is scarce. Sharing of global experience on health systems teaching will strengthen the field, recognising the limitations of small programs and strengths of larger, more experienced programs. As “health systems” is entrenched in global health jargon, a risk remains that imprecise definition and a lack of consensus will constrain efforts to build the skills necessary to improve public health delivery [22].

Frenk et al. called “on the most important constituencies to embrace the imperative for reform through dialogue, open exchange, discussion, and debate” [17]. The international public health community in Australia is responding to this call and cross-university dialogue to enhance health systems education is underway. Sharing and reflecting on Australian experience reveals a diversity of perspectives and lessons that deserve further documentation, analysis and evaluation, if teaching and understanding in this complex area is to be enhanced.

## Abbreviations

MPH: Masters of Public Health; MIPH: Masters of International Public Health; LMIC: Low and Middle Income Countries.

## Competing interests

There are no financial conflicts of interest. The authors have drawn upon examples from the institutions in which they are based and from courses which they teach.

JN is partly funded by an Australian National Health and Medical Research Council Health Economics Capacity Building Grant.

AM is partly funded by a University of Sydney Fellowship.

Burnet Institute (CM) acknowledges the support of the Victorian Operational Infrastructure Support Program.

The funders had no role in study design, data collection and analysis, decision to publish, or preparation of the manuscript.

## Authors’ contributions

JN and AM conceived of the paper and wrote the first draft. All authors provided input into content and edited the draft. All authors read and approved the final manuscript.

## Pre-publication history

The pre-publication history for this paper can be accessed here:

http://www.biomedcentral.com/1472-698X/12/14/prepub

## References

[B1] Gwatkin DR Bhuiya A Victora CG Making health systems more equitable Lancet 2004 364 1273 1280 10.1016/S0140-6736(04)17145-6 15464189

[B2] World Health Organization World Report on Better Knowledge for Health: Strengthening Health Systems 2004 World Health Organization, Geneva

[B3] Travis P Bennett S Haines A Pang T Bhutta Z Hyder AA Pielemeier NR Mills A Evans T Overcoming health-systems constraints to achieve the Millennium Development Goals Lancet 2004 364 900 906 10.1016/S0140-6736(04)16987-0 15351199

[B4] Task Force on Health Systems Research Informed choices for attaining the Millennium Development Goals: towards an international cooperative agenda for health-systems research Lancet 2004 364 997 1003 15364193 10.1016/S0140-6736(04)17026-8

[B5] World Health Organization Everybody’s Business: Strengthening Health Systems to Improve Health Outcomes – WHO’s Framework for Action 2007 World Health Organization, Geneva

[B6] USAID Sustaining health gains: building systems USAID Report to Congresshttp://www.usaid.gov/our_work/global_health/hs/publications/hss_report.html

[B7] World Bank Health development: the World Bank strategy for health, nutrition and population results 2007 World Bank, Washington, DC

[B8] Marchal B Cavalli A Kegels G Global Health Actors Claim To Support Health System Strengthening—Is This Reality or Rhetoric? PLoS Med 2009 6 4 e1000059 10.1371/journal.pmed.1000059 19399158 PMC2667637

[B9] Reich MR Takemi K Roberts MJ Hsiao WC Global action on health systems: a proposal for the Toyako G8 summit Lancet 2008 371 865 869 10.1016/S0140-6736(08)60384-0 18328932

[B10] Institute of Medicine: Committee on Health Services Research Institute of Medicine Committee on Health Services Research: Training and Work Force Issues Health Services Research: Workforce and Educational Issues 1995 National Academy Press, Washington, DC

[B11] Roberts M Hsiao W Berman P Reich M Getting Health Reform Right: a guide to improving performance and equity 2003 Oxford University Press, New York

[B12] Atun R De Jongh T Secci K Ohiri K Adeyi O Integration of targeted health interventions into health systems: a conceptual framework for analysis Health Policy and Planning 2009 24 1 8 19917651 10.1093/heapol/czp055

[B13] Shakarishvili G Atun R Berman P Hsiao W Burgess C Lansang MA Converging health systems frameworks: towards a concepts-to-actions roadmap for health systems strengthening in low and middle income countries Global Health Governance 2010 3 2 1 622506090

[B14] Swanson RC Bongiovanni A Bradley E Murugan V Sundewall J Betigeri A Nyonator F Cattaneo A Harless B Ostrovsky A Labonté R Toward a Consensus on Guiding Principles for Health Systems Strengthening PLoS Med 2010 7 12 e1000385 10.1371/journal.pmed.1000385 21203584 PMC3006350

[B15] Shakarishvili G Lansang MA Mitta V Bornemisza O Blakley MG Lansang MA Mitta V Bornemisza O Blakley M Kley N Burgess C Atun R Health systems strengthening: a common classification and framework for investment analysis Health Policy and Planning 2011 26 4 316 2 10.1093/heapol/czq053 20952397 PMC3118911

[B16] Sterman J Learning from evidence in a complex world Am J Public Health 2006 96 3 505 14 10.2105/AJPH.2005.066043 16449579 PMC1470513

[B17] Frenk J Chen L Bhutta ZA Cohen J Crisp N Evans T Fineberg H Garcia P Ke Y Kelley P Kistnasamy B Meleis A Naylor D Pablos-Mendez A Reddy S Scrimshaw S Sepulveda J Serwadda D Zurayk H Health professionals for a new century: transforming education to strengthen health systems in an interdependent world Lancet 2010 376 1923 1958 10.1016/S0140-6736(10)61854-5 21112623

[B18] Evans D The role of schools of public health: learning from history, looking to the future Journal of Public Health 2009 31 3 446 450 10.1093/pubmed/fdp065 19574273

[B19] Sadana R Mushtaque A Chowdhury R Petrakova A Strengthening public health education and training to improve global health Bull World Health Organ 2007 85 3 163 4 10.2471/BLT.06.039321 17486201 PMC2636237

[B20] Merson M Health Systems Strengthening through Human Resources and Capacity Building: A Landscaping Study on the Supply of Health Systems Training Programs and the Demand for Health Systems Experts Duke Global Health Institute 2008 Strengthening Health Systems Capacity and Leadership, Bellagio Conference October 28 http://www.columbia-icap.org/bellagio2/LHSSI%20Oct%2019%20PO.pdf

[B21] Bennett S Paina L Kim C Agyepong I Chunharas S McIntyre D Nachuk S What must be done to enhance capacity for Health Systems Research? Background paper for the global symposium on health systems research 2010 World Health Organization, Montreux, Switzerland 16–19 November

[B22] Bennett S Agyepong IA Sheikh K Hanson K Ssengooba F Gilson L Building the field of health policy and systems research: an agenda for action PLoS Med 2011 8 8 e1001091 21918641 10.1371/journal.pmed.1001081 PMC3168867

[B23] Beaglehole R Dal Poz MR Public health workforce: challenges and policy issues Hum Resour Heal 2003 1 4 10.1186/1478-4491-1-4 PMC179882 12904251

[B24] Consortium of Universities for Global Health Universities report doubling of students enrolled in global health programs in last 3 yearshttp://www.cugh.org/sites/default/files/press-release-sept-14-2009.pdf

[B25] Association of Schools of Public Health Global Health Competency Model DRAFT Model Version 1.0 http://www.asph.org/userfiles/Narrative&GraphicGHCompsVersion1.0_2011-08-26.pdf

[B26] Akbar H Hill PS Rotem A Riley ID Zwi AB Marks GC Mark T Identifying competencies for Australian health professionals working in international health Asia Pacific Journal of Public Health 2005 17 2 99 103 10.1177/101053950501700207 16425653

[B27] Sheikh K Gilson L Agyepong IA Hanson K Ssengooba F Bennett S Building the field of Health Policy and Systems Research: framing the questions PLoS Med 2011 8 8 e1001073 10.1371/journal.pmed.1001073 21857809 PMC3156683

[B28] Ruxin J Doctors without Orders Democracy Journal 2008 9 32 43

